# Temporal dynamics of the mimetic allele frequency at the *doublesex* locus, which controls polymorphic Batesian mimicry in *Papilio memnon* butterflies

**DOI:** 10.1038/s41598-017-13419-8

**Published:** 2017-10-10

**Authors:** Shinya Komata, Chung-Ping Lin, Teiji Sota

**Affiliations:** 1 Department of Zoology, Graduate School of Science, Kyoto University, Kyoto, Japan; 2 Department of Life Science, National Taiwan Normal University, Taipei, Taiwan

## Abstract

Tracking allele frequencies is essential for understanding how polymorphisms of adaptive traits are maintained. In *Papilio memnon* butterflies, which exhibit a female-limited Batesian mimicry polymorphism (wing-pattern polymorphism), two alleles at the *doublesex* (*dsx*) locus correspond to mimetic and non-mimetic forms in females; males carry both *dsx* alleles but display only the non-mimetic form. This polymorphism is thought to be maintained by a negative frequency-dependent selection. By tracking *dsx* allele frequencies in both sexes at a Taiwanese site over four years, we found that the mimetic allele persists at intermediate frequencies even when the unpalatable model papilionid butterflies (*Pachliopta* and *Atrophaneura* species) were very rare or absent. The rates of male mate choice did not differ between the two female forms; neither did insemination number nor age composition, suggesting equivalent reproductive performance of the two forms over time. Our results characterised the temporal dynamics of the mimetic allele frequency in the field for the first time and give insights into underlying processes involved in the persistence of the female-limited Batesian mimicry polymorphism.

## Introduction

Batesian mimicry is a textbook example of evolution by natural selection^1,2^. In Batesian mimicry, palatable mimics avoid predation by resembling unpalatable models^1^. The advantage of this mimicry is negatively frequency-dependent, decreasing when the relative abundance of Batesian mimics to unpalatable models is high because of quick learning by predators^2,3^. The most intriguing phenomenon regarding Batesian mimicry in butterflies is the female-limited polymorphism, in which females display both mimetic and non-mimetic forms, but males are non-mimetic^4,5^. Female-limited mimicry is attributed to stronger predation pressure upon females relative to males, resulting in a greater fitness advantage of mimicry only in females^4–7^. Moreover, butterfly colouration may be subject to sexual selection (e.g., female choice^8^ and male-male competition^9^), which may constrain the evolution of mimicry in males despite potential benefits to both sexes. Female-limited mimicry polymorphisms in butterflies are thought to be maintained by negative frequency-dependent selection (NFDS) acting on different female wing patterns, in which the advantage of mimetic forms decreases as their frequency increases^5,10,11^. To understand the mechanism of maintenance for mimicry polymorphisms, it is necessary to study the dynamics of mimic, model and predator populations while monitoring changes in the frequency of mimicry-related alleles. However, field-based population genetic studies of mimicry polymorphisms are challenging due to a lack of knowledge about the genetic basis of the polymorphism.

Recent genomic studies of *Papilio polytes* ^12,13^ have revealed that mimetic/non-mimetic phenotypes are controlled by two alleles at the *doublesex* (*dsx*) locus, which corresponds to the hypothesised supergene that controls female phenotypes in a Mendelian fashion; the mimetic allele *H* is dominant to the non-mimetic allele *h* ^14–16^. We demonstrated that *P*. *memnon* also possesses similar alleles that strictly control female phenotypes^17^. These findings provide a simple genotyping method for studying temporal changes in mimetic allele frequencies in the field^17^. More importantly, we can obtain the allele frequency in a population directly from males, which are captured more frequently than females due to behavioural or other factors^7,18,19^. Males exhibit a monotypic wing pattern that differs from that of females regardless of the *dsx* genotypes; thus, the allele frequencies in males should be equivalent to those in emerging (pre-selective) females of a given generation.

Here, we report the results of a 4-year study of the temporal dynamics of mimetic/non-mimetic alleles in *P*. *memnon* in relation to the temporal abundance of models. Our objective was to elucidate how the female-limited Batesian mimicry polymorphism is maintained over time. We also compared the life span, lifetime mating number (number of spermatophores) and male mate choice between mimetic and non-mimetic females in the field to assess whether these fitness components vary with phenotype.

## Results and Discussion

**Figure 1:**
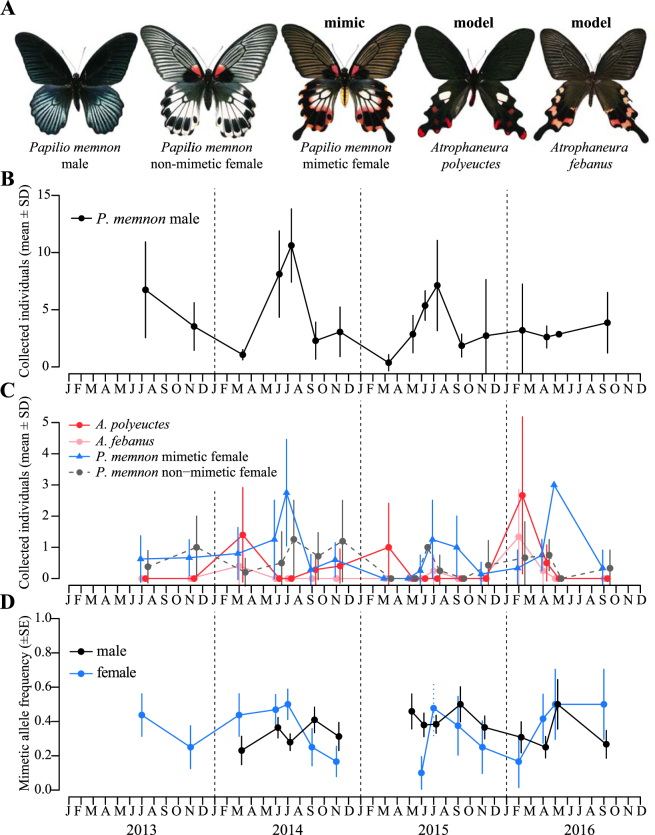
Results of the field study in Hualien, eastern Taiwan. (**A**) *Papilio memnon* and its model species, *Atrophaneura febanus* and *Atrophaneura polyeuctes*. (**B**) Mean number of *P*. *memnon* males collected per line-transect census. (**C**) Mean numbers of individuals of the Batesian mimicry models *A*. *febanus* and *A*. *polyeuctes* and mimetic *P*. *memnon* females collected per line-transect census. Another model, *Pachliopta aristolochiae*, was collected only once, in July 2013, and is not shown. (**D**) Frequencies of the *doublesex H* allele (mimetic allele) in males and females of *P*. *memnon*. Genomic DNA extraction failed for females in July 2015; therefore, the min-max (dotted vertical line) and intermediate values of the *H* allele frequency were estimated from the numbers of mimetic and non-mimetic females collected.

Line-transect censuses of butterflies were conducted 17 times at our field site in eastern Taiwan from 2013 to 2016. During this period, we collected butterflies of 11 papilionid species, including 2 Batesian mimicry species, *P*. *memnon* (307 males, 50 mimetic females and 39 non-mimetic females) and *P*. *polytes* (38 males, 11 mimetic females and 5 non-mimetic females), and three unpalatable models, *Atrophaneura polyeuctes* (*n* = 21), *Atrophaneura febanus* (*n* = 8) and *Pachliopta aristolochiae* (*n* = 1)^15,16^ (Figs 1A and S1 and Table S1, Supplementary Information). Thus, *P*. *memnon* was the most abundant species, followed by *P*. *polytes*, whereas model species were substantially less abundant. Although we collected almost all *Papilio* butterflies encountered, males were more abundant than females in the samples (male:female ratios, *P*. *memnon* = 1:0.29; *P*. *polytes* = 1:0.42; Table S1), as is often the case in butterflies^7,17–19^. We collected *P*. *memnon* during all 17 samplings, although they were most abundant in summer (June–July) (Fig. 1B; Tables S1 and S4, Supplementary Information). The model species were relatively abundant in February–March but were otherwise rare. Thus, mimetic *P*. *memnon* females were more abundant than the model species throughout the year, except during February and March (Fig. 1C).

We genotyped the *dsx* locus for 303 males and 99 females of *P*. *memnon* (Table S2, Supplementary Information). The genotype frequencies did not depart from the expectations of Hardy-Weinberg equilibrium at any of the sampling times (Table S2). The frequency of the *H* allele at each sampling time ranged between 0.23 and 0.5 for males (average, 0.35) and between 0.1 and 0.5 for females (average, 0.37) (Fig. 1D). The estimated frequency was more reliable for males than females because of larger sample sizes. We found no predictable pattern of fluctuations in the frequency of the *H* allele in males although the frequency did fluctuate throughout the course of the study (Table S5, Supplementary Information).

To see whether the *H* allele frequency was controlled by NFDS, we analysed the relationship between the *H* allele frequencies in male and female populations at each sampling time. We assumed that the *H* allele frequency was identical between the sexes at the time of adult emergence but differential selection on the female forms caused changes in the *H* allele frequency sometime after emergence only in females; the allele frequency in males, which show no mimetic wing patterns or wing pattern polymorphisms, should remain constant (in fact wing wear data suggested that our samples consisted mainly of aged individuals; see below). We examined whether the *H* allele frequency differed between the sexes in each sampling time but found no significant case (12 sampling times; Fisher’s exact test, *P* = 0.042–1; not significant after Bonferroni correction). Thus, we could not show differential selection on the two female forms, although this result might be largely affected by insufficient sample sizes in the present data.

In *P*. *memnon*, age structure (estimated by wing wear) did not significantly differ among males, mimetic females and non-mimetic females (Table 1). The forewing lengths of females were significantly longer than those of males but did not differ between mimetic and non-mimetic forms (Fig. S2, Supplementary Information; Table S4). Forewing lengths in February–April were shorter than in other months in both sexes and both female forms (Fig. S2; Table S4). Older females contained more spermatophores in their bursa copulatrix regardless of their form (Tables 2 and S5). We found no evidence of a male preference for either of the female forms (male choices for mimetic and non-mimetic forms, 27:29; binomial test; *P* = 0.89; see also Tables S3 and S5, Supplementary Information). Therefore, the two female forms did not appear to differ in life span, wing size, lifetime mating number or mating opportunities via male mate choice. The lack of differences between the two female forms suggests that they can attain similar levels of fitness if the predation pressure is equal. We did not directly examine differences in predation between forms, but we did assess the occurrence of beak marks on wings (an indicator of bird attack^7^; but see Burger & Gochfeld^20^ for criticism of the use of this indicator). Although the probability of having beak marks increased with age, the two female forms did not differ (Tables 1 and S5). Therefore, predation pressure on both forms appeared to be similar when averaged over several generations.

**Table 1:** Age structure and percentage of butterflies with beak marks on their wings in *Papilio memnon*.

Age	Male			Mimetic female			Non-mimetic female		
	*n*	BM	BM (%)	*n*	BM	BM (%)	*n*	BM	BM (%)
1	82	15	18.3	15	3	20.0	5	0	0
2	190	111	58.4	35	22	62.9	28	20	64.5
3	88	82	93.2	16	13	81.3	9	9	90.0
Total	360	208	57.8	66	38	57.6	42	29	69.0

The age structure among males, mimetic females and non-mimetic females did not differ significantly (Fisher’s exact probability test, *P* = 0.4787). The beak mark rate increased with age (generalised linear mixed model [GLMM] with a binomial error: slope ± s. e. = 1.992 ± 0.2123) regardless of form (Table S5, Supplementary Information).

**Table 2:** Number of spermatophores in *Papilio memnon* females collected in the field.

Form	Age	*n*	No. of spermatophores					Mean no. of spermatophores (±SD)
			0	1	2	3	4	
Mimetic female	1	15	1	14	0	0	0	0.933 ± 0.26
	2	35	1	21	10	3	0	1.429 ± 0.70
	3	16	1	2	80	5	0	2.063 ± 0.85
Total		66						
Non mimetic female	1	5	1	4	0	0	0	0.800 ± 0.45
	2	28	0	19	7	1	1	1.429 ± 0.74
	3	9	0	4	4	0	1	1.778 ± 0.97
Total		42						

The number of spermatophores increased with age (generalised linear mixed model [GLMM] with Poisson error: slope ± s. e. = 0.3686 ± 0.1257) regardless of form (Table S5, Supplementary Information).

We found that the abundance of model species was relatively low throughout the year, except in early spring (Fig. 1). Although the condition of fewer models than mimics would hinder the evolution of monotypic Batesian mimicry and hence favor polymorphisms, such a condition may also diminish the protection offered by Batesian mimicry^21–23^. At our study site, the abundance of models peaked in spring (February–March) but declined to levels much lower than the abundance of mimetic *P*. *memnon* females by summer (Fig. 1C). A similar phenological trend of a model species, *A*. *polyeuctes*, has been reported in southern Taiwan^24^. The different phonological trends of these papilionids may be related to the difference in larval host plants; *P*. *memnon* uses wild and cultivated trees of Rutaceae, whereas the model species (*A*. *polyeuctes* and *A*. *febanus*) woody vines of *Aristolochia* (Aristolochiaceae). Although the reversal of the relative abundance of mimics to models could be disadvantageous for mimics, the frequency of the mimetic allele did not decrease in the absence of models during these times (Fig. 1C,D; Table S5). The advantage of mimicry may persist until later seasons due to the efficacy of predator learning in the spring. In addition, the presence of abundant alternative prey in later seasons may allow for the persistence of mimics with low model abundance. The availability of alternative prey has been shown to alter model-mimic relationships^25^. Alternatively, the persistence of the mimetic form despite a low abundance of models may be more fundamentally caused by the strong psychological impact of toxic models on bird foraging behaviour. A recent avian study revealed that birds tend to avoid prey types with variable toxicity^26^, implying that birds will avoid mimics even when the encounter rate of highly unpalatable models is lower than that of palatable mimics. Therefore, the predation risk for mimics will remain low unless the relative abundance of mimics to models becomes very high. Our results support the previously reported evidence that model abundance does not have to be high for Batesian mimicry to persist^27–29^.

In our previous study, we found that the mimetic allele frequency in *P*. *memnon* in July at nine localities throughout Taiwan ranged between 0.36 and 0.55^17^. Together with the present results, these data indicate that the mimicry polymorphism of *P*. *memnon* appears to persist at moderate levels of the mimetic allele frequency both spatially and temporally in Taiwan. The precise mechanism of the allelic frequency change is unclear, but it is possibly caused by negatively frequency-dependent predation on different female phenotypes. Additional studies of predator behaviour and the mortality processes of the two female forms are needed to understand the selection pressures acting on the wing patterns of *P*. *memnon* butterflies.

## Methods

### Line-transect census of wild butterflies

We conducted 71 line-transect censuses in mixed habitats, including a pomelo (*Citrus maxiama*) orchard and a wasteland at Hualien, eastern Taiwan (23°59′N, 121°32′E; elevation: 565 m) over 17 time periods from July 2013 to September 2016. At each census, a researcher (SK) walked both directions of a fixed route (1000 m) along a road for 1 h, collecting all papilionid butterflies observed. In addition, all male and female *P*. *memnon* butterflies seen outside of the hour-long census periods were collected.

### Genotype and allele frequencies

Total genomic DNA was extracted from the legs or thorax of *P*. *memnon* using a Genomic DNA Purification Kit (Promega, Madison, WI, USA). High-Resolution Melting analysis using a real-time PCR platform was performed to identify *dsx* genotypes following Komata *et al*.^17^. The proportions of different *dsx* genotypes in each month were evaluated in terms of Hardy-Weinberg equilibrium using the R ^30^ package HardyWeinberg^31^. See Table S2 (Supplementary Information) for more information.

### Age structure, body size, beak mark rate and lifetime mating number

Age was estimated based on wing wear rankings: 1, little or no wing wear; 2, moderate wing wear and 3, heavy wing wear. Variations among ages in males, mimetic females and non-mimetic females were analysed using Fisher’s exact probability test. Forewing length was measured using electronic digital callipers except for individuals with severely damaged forewings. Marks larger than 5 mm in length on butterfly wings were recorded as “beak marks” by bird attacks^7^. We dissected the bursa copulatrix from *P*. *memnon* females to count the number of spermatophores, which is an indication of the number of times the female has mated.

### Male mate choice experiment

Experiments were conducted on 9–12 June and 15–17 July, 2014, at the same site as the line-transect censuses. A pair of mimetic and non-mimetic specimens was displayed in the foliage of pomelo trees in June, while in July three pairs were displayed in the same way at 3–4-m intervals. The distance between the two specimens was about 40 cm, and their positions were changed daily. The experiment was conducted in the morning (5:30–10:00), when males actively search out females for mating. After confirming courtship behaviour to either specimen, males were collected using an insect net to avoid double counts and for later analysis of their *dsx* genotypes.

### Statistical analyses

We explored the effect of sampling season (season) on the number of *P*. *memnon* males collected per census using a generalised linear mixed model (GLMM) assuming Poisson distribution. Each year was divided into three sampling seasons (January–April, May–August and September–December) for all analyses. The sampling year (year) was included as a random effect. Forewing length was analysed with a GLMM assuming a Gaussian distribution with season and sex/form as fixed effects, and year as a random effect.

In analyses using *dsx* mimetic allele frequencies in males and females, beak mark rate, and the number of spermatophores, we performed GLMM analyses with Akaike’s Information Criterion corrected for small sample size (AICc) to determine the model with the optimal set of explanatory variables. The *dsx* mimetic allele frequencies in males and females were analysed with a GLMM assuming a binomial distribution with season and the number of models (total number of *A*. *polyeuctes*, *A*. *febanus* and *Pachliopta aristolochiae* collected in the field) as fixed effects, and year as a random effect. The beak mark rate (individuals with beak marks/total number of individuals) was analysed using a GLMM with a binomial distribution, with season, forewing length, age and sex/form as fixed effects. The number of spermatophores was analysed using a Poisson distribution with forewing length, age, and sex/form as fixed effects. The sampling time period (n = 16) was treated as a random effect for analyses of the beak mark rate and the number of spermatophores. For mate choice, the selection of mimetic/non-mimetic specimens was analysed using a binomial test, after confirming the effects of male genotype and experimental settings with a generalised linear model analysis assuming a binomial distribution with AICc to determine the model with the optimal set of explanatory variables. All analyses were performed using R^30^ packages lme4^32^ and MuMIn^33^.

## Electronic supplementary material

Supplementary Information
