# Supplementary material for: Temporal dynamics of the mimetic allele frequency at the doublesex locus, which controls polymorphic Batesian mimicry in Papilio memnon butterflies
Source: Sci Rep. 2017 Oct 10;7:12926. doi: 10.1038/s41598-017-13419-8 (PMC5635110; doi:10.1038/s41598-017-13419-8)
Supplement: Supplementary file 1 — Supplementary Information [file 41598_2017_13419_MOESM1_ESM.doc]

**Supplementary Information for:**

**Temporal dynamics of the mimetic allele frequency at the *doublesex* locus, which controls polymorphic Batesian mimicry in *Papilio memnon* butterflies**

Shinya Komata^1^, Chung-Ping Lin^2^ & Teiji Sota^1^

^1^Department of Zoology, Graduate School of Science, Kyoto University, Kyoto, Japan.

^2^Department of Life Science, National Taiwan Normal University, Taipei, Taiwan.

**Figure S1.** Mean numbers of males, mimetic females and non-mimetic females of *Papilio* *polytes* collected per line-transect census.

**Figure S2.** Forewing length of males, mimetic females and non-mimetic females of *Papilio* *memnon* collected each month. The gray block shows the summer season. The yearly sampling period was divided into three seasons (January–April, May–August and September–December).

**Table S1.** Results of the line-transect census in Hualien, eastern Taiwan.

|  | No. censuses | *Papilio memnon* | | |  | *Papilio polytes* | | | *A_p_* ^‡^ | *A_f_* ^‡^ | *P_a_* ^‡^ |
| --- | --- | --- | --- | --- | --- | --- | --- | --- | --- | --- | --- |
|  |  | *n*_male_ | *n*_m.f_. ^†^ | *n*_nm.f_. ^†^ |  | *n*_male_ | *n*_m.f_. ^†^ | *n*_nm.f_. ^†^ |  |  |  |
| 2013 |  |  |  |  |  |  |  |  |  |  |  |
| 27, 28 July | 8 | 55 | 5 | 3 |  | 15 | 2 | 1 | 0 | 0 | 1 |
| 9, 10 November | 3 | 11 | 2 | 3 |  | 0 | 0 | 1 | 0 | 0 | 0 |
| 2014 |  |  |  |  |  |  |  |  |  |  |  |
| 25, 26, 28 March | 5 | 6 | 4 | 1 |  | 2 | 1 | 0 | 7 | 2 | 0 |
| 9-12 June | 4 | 33 | 5 | 2 |  | 4 | 2 | 0 | 0 | 0 | 0 |
| 14-17 July | 4 | 43 | 11 | 5 |  | 9 | 1 | 2 | 0 | 0 | 0 |
| 15-18 September | 7 | 17 | 2 | 5 |  | 2 | 2 | 0 | 2 | 0 | 0 |
| 15-17 November | 5 | 16 | 3 | 6 |  | 0 | 0 | 0 | 2 | 0 | 0 |
| 2015 |  |  |  |  |  |  |  |  |  |  |  |
| 9, 10 March | 2 | 1 | 0 | 0 |  | 0 | 0 | 0 | 2 | 0 | 0 |
| 13-15 May | 4 | 12 | 0 | 0 |  | 2 | 0 | 0 | 0 | 0 | 0 |
| 6-8 June | 4 | 22 | 1 | 4 |  | 2 | 0 | 0 | 0 | 0 | 0 |
| 6-8 July | 4 | 29 | 5 | 1 |  | 1 | 0 | 0 | 0 | 1 | 0 |
| 29-30 September | 3 | 6 | 3 | 0 |  | 0 | 0 | 0 | 0 | 0 | 0 |
| 10-11 November | 7 | 20 | 1 | 3 |  | 0 | 0 | 0 | 0 | 0 | 0 |
| 2016 |  |  |  |  |  |  |  |  |  |  |  |
| 18, 19 February | 3 | 10 | 1 | 2 |  | 0 | 2 | 0 | 8 | 4 | 0 |
| 7-9 April | 4 | 11 | 3 | 3 |  | 0 | 0 | 0 | 2 | 1 | 0 |
| 15 May | 1 | 3 | 3 | 0 |  | 0 | 0 | 0 | 0 | 0 | 0 |
| 13, 16 September | 3 | 12 | 1 | 1 |  | 1 | 1 | 1 | 0 | 0 | 0 |
|  |  |  |  |  |  |  |  |  |  |  |  |
| Total | 71 | 307 | 50 | 39 |  | 38 | 11 | 5 | 23 | 8 | 1 |

^†^ “*n*_m.f._” and “*n*_nm.f._” are the numbers of mimetic and non-mimetic females, respectively.

^‡^ “*A_p_*”, “*A_f_*” and “*P_a_*” are the numbers of *Atrophaneura polyeuctes*, *Atrophaneura febanus* and *Pachliopta aristolochiae*, respectively.

**Table S2.** Number of samples analysed and the *doublesex* allele types in *Papilio* *memnon* individuals collected in the field.

|  | Male | | | | |  | Female | | | | |
| --- | --- | --- | --- | --- | --- | --- | --- | --- | --- | --- | --- |
|  | *n* | *HH* | *Hh* | *hh* | *P*^†^ |  | *n* | *HH* | *Hh* | *hh* | *P*^†^ |
| 2013 |  |  |  |  |  |  |  |  |  |  |  |
| July | (50)^‡^ | - | - | - | - |  | 8 | 2 | 3 | 3 | 0.53 |
| November | (12)^‡^ | - | - | - | - |  | 6 | 0 | 3 | 3 | 1.00 |
| 2014 |  |  |  |  |  |  |  |  |  |  |  |
| March | 13 | 0 | 6 | 7 | 1.00 |  | 8 | 0 | 7 | 1 | 0.14 |
| June | 33 | 4 | 16 | 13 | 1.00 |  | 16 | 3 | 9 | 4 | 1.00 |
| July | 43 | 3 | 18 | 22 | 1.00 |  | 16 | 5 | 6 | 5 | 0.34 |
| September | 22 | 4 | 10 | 8 | 1.00 |  | 8 | 1 | 2 | 5 | 0.38 |
| November | 16 | 1 | 8 | 7 | 1.00 |  | 9 | 0 | 3 | 6 | 1.00 |
| 2015 |  |  |  |  |  |  |  |  |  |  |  |
| March | 1 | - | - | - | - |  | 0 | - | - | - | - |
| May | 12 | 1 | 9 | 2 | 0.24 |  | 0 | - | - | - | - |
| June | 25 | 5 | 9 | 11 | 0.23 |  | 5 | 0 | 1 | 4 | 1.00 |
| July | 43 | 5 | 23 | 15 | 0.53 |  | (11)^‡^ | (6) | | (5) | - |
| September | 12 | 3 | 6 | 3 | 1.00 |  | 4 | 0 | 3 | 1 | 1.00 |
| November | 26 | 4 | 11 | 11 | 0.68 |  | 4 | 1 | 0 | 3 | 0.14 |
| 2016 |  |  |  |  |  |  |  |  |  |  |  |
| February | 13 | 1 | 6 | 6 | 1.00 |  | 3 | 0 | 1 | 2 | 1.00 |
| April | 24 | 1 | 10 | 13 | 1.00 |  | 6 | 1 | 3 | 2 | 0.94 |
| May | 6 | 1 | 4 | 1 | 1.00 |  | 3 | 0 | 3 | 0 | 0.40 |
| September | 15 | 1 | 6 | 8 | 1.00 |  | 3 | 1 | 1 | 1 | 1.00 |
|  |  |  |  |  |  |  |  |  |  |  |  |
| Total |  | 34 | 142 | 127 | 0.61 |  |  | 14 | 45 | 40 | 0.83 |

^†^ *P*-values from Fisher’s exact probability test for Hardy-Weinberg equilibrium.

^‡^ The *doublesex* allele types of these samples were unknown, as genomic DNA extraction was unsuccessful. These samples were excluded from related analyses.

**Table S3.** Number of males choosing mimetic or non-mimetic females in mate-choice experiments.

|  | *HH* ^†^ | |  | *Hh* ^†^ | |  | *hh* ^†^ | |  | total | |  |
| --- | --- | --- | --- | --- | --- | --- | --- | --- | --- | --- | --- | --- |
|  | M ^‡^ | NM ^‡^ |  | M | NM |  | M | NM |  | M | NM |  |
| June |  |  |  |  |  |  |  |  |  |  |  |  |
| Pair 1 | 1 | 1 |  | 2 | 9 |  | 6 | 5 |  | 9 | 15 |  |
| July |  |  |  |  |  |  |  |  |  |  |  |  |
| Pair 1 | 0 | 0 |  | 1 | 3 |  | 3 | 3 |  | 4 | 6 |  |
| Pair 2 | 1 | 1 |  | 3 | 1 |  | 4 | 1 |  | 8 | 3 |  |
| Pair 3 | 2 | 0 |  | 3 | 1 |  | 1 | 1 |  | 6 | 2 |  |
| Total |  |  |  |  |  |  |  |  |  | 27 | 26 |  |

^†^ The *doublesex* genotypes of males used in the experiment. ^‡^ “M” and “NM” are the numbers of males choosing specimens of mimetic and non-mimetic females, respectively. Model selection in binomial generalized linear model based on Akaike’s Information Criterion corrected for small sample size (AICc) identified the pair of specimens as an explanatory variable for determining male mate preference (Table S5). The preferences were slightly biased toward mimetic females in Pair 2 (generalised linear model [GLM] with a binomial error: slope ± s. e. = 1.4604 ± 0.7635) and Pair 3 (slope ± s. e. = 1.5782 ± 0.8895), but were biased toward non-mimetic females in Pair 1.

**Table S4.** Results of generalised linear mixed model (GLMM) analyses on the number of males collected per census and the forewing length in *Papilio* *memnon*.

|  | Estimate | Error | *z*/*t* | *P* |
| --- | --- | --- | --- | --- |
| **The number of *P*. *memnon* males collected per census** | | | | |
| Intercept | 0.6368 | 0.2091 | 3.045 | 0.0023 |
| Season [May-August] | 1.3132 | 0.2196 | 5.980 | < 0.0001 |
| Season [September-December] | 0.4427 | 0.2282 | 1.940 | 0.052 |
|  |  |  |  |  |
| **Forewing length** |  |  |  |  |
| Intercept | 67.273 | 0.6440 | 104.463 | < 0.0001 |
| Season [May-August] | 5.258 | 0.4528 | 11.611 | < 0.0001 |
| Season [September-December] | 3.778 | 0.4610 | 8.195 | < 0.0001 |
| Sex/Form [mimetic female] | 0.086 | 0.5378 | 0.160 | 0.873 |
| Sex/Form [male] | -4.675 | 0.4478 | -10.439 | < 0.0001 |

The sampling year was included as a random effect in each model.

**Table S5.** Model selection of generalised linear model (GLM) and generalised linear mixed model (GLMM) analyses incorporating *doublesex* (*dsx*) mimetic allele frequencies, beak mark rate, number of spermatophores and male mate preference in *Papilio memnon*.

| models | d.f. | LogLik | AICc | ∆AICc | weight |
| --- | --- | --- | --- | --- | --- |
| **GLMM: The dsx mimetic allele frequencies in male** | | | | | |
| intercept-only model (null model) | 2 | -34.265 | 73.6 | 0.00 | 0.666 |
| unpalatable models | 3 | -33.739 | 75.9 | 2.26 | 0.215 |
| season | 4 | -32.404 | 77.3 | 3.63 | 0.108 |
| season, unpalatable models | 5 | -32.236 | 82 | 8.35 | 0.01 |
| **GLMM: The dsx mimetic allele frequencies in female** | | | | | |
| intercept-only model (null model) | 2 | -27.554 | 60.2 | 0.00 | 0.596 |
| season | 4 | -24.926 | 62.3 | 2.10 | 0.209 |
| unpalatable models | 3 | -27.238 | 62.9 | 2.68 | 0.156 |
| season, unpalatable models | 5 | -24.092 | 65.7 | 5.49 | 0.038 |
| **GLMM: Beak mark rate** |  |  |  |  |  |
| age | 3 | -248.428 | 502.9 | 0.00 | 0.388 |
| forewing, age | 4 | -247.688 | 503.5 | 0.55 | 0.294 |
| age, sex/form | 5 | -247.793 | 505.7 | 2.81 | 0.095 |
| age, season | 5 | -247.885 | 505.9 | 2.99 | 0.087 |
| forewing, age, sex/form | 6 | -247.282 | 506.7 | 3.84 | 0.057 |
| forewing, age, season | 6 | -247.490 | 507.2 | 4.25 | 0.046 |
| age, season, sex/form | 7 | -247.194 | 508.6 | 5.72 | 0.022 |
| forewing, age, season, sex/form | 8 | -247.023 | 510.4 | 7.45 | 0.009 |
| forewing | 3 | -312.095 | 630.2 | 127.34 | 0 |
| season | 4 | -311.232 | 630.5 | 127.64 | 0 |
| forewing, season | 5 | -310.557 | 631.2 | 128.34 | 0 |
| intercept-only model (null model) | 2 | -313.628 | 631.3 | 128.38 | 0 |
| season, sex/form | 6 | -309.950 | 632.1 | 129.17 | 0 |
| forewing, sex/form | 5 | -311.183 | 632.5 | 129.59 | 0 |
| sex/form | 4 | -312.502 | 633.1 | 130.18 | 0 |
| forewing, season, sex/form | 7 | -309.633 | 633.5 | 130.60 | 0 |
| **GLMM: Spermatophore** |  |  |  |  |  |
| age | 3 | -136.494 | 279.2 | 0.00 | 0.524 |
| forewing, age | 4 | -136.435 | 281.3 | 2.04 | 0.189 |
| age, sex/form | 4 | -136.45 | 281.3 | 2.07 | 0.186 |
| forewing, age, sex/form | 5 | -136.393 | 283.4 | 4.16 | 0.066 |
| intercept-only model (null model) | 2 | -140.847 | 285.8 | 6.59 | 0.019 |
| forewing | 3 | -140.77 | 287.8 | 8.55 | 0.007 |
| sex/form | 3 | -140.832 | 287.9 | 8.68 | 0.007 |
| forewing, sex/form | 4 | -140.757 | 289.9 | 10.68 | 0.003 |
| **GLM: Male mate preference** |  |  |  |  |  |
| pair | 3 | -33.561 | 73.6 | 0.00 | 0.458 |
| intercept-only model (null model) | 1 | -36.727 | 75.5 | 1.92 | 0.175 |
| genotype, pair | 5 | -32.296 | 75.9 | 2.26 | 0.148 |
| genotype | 3 | -35.514 | 77.5 | 3.91 | 0.065 |
| pair, possition | 7 | -30.567 | 77.6 | 4.01 | 0.062 |
| possition | 6 | -32.08 | 78 | 4.37 | 0.051 |
| genotype, possition | 8 | -30.224 | 79.7 | 6.11 | 0.022 |
| genotype, pair, possition | 9 | -28.85 | 79.9 | 6.27 | 0.02 |

d. f., degrees of freedom; LogLik, log-likelihood; AICc, AIC corrected for small sample size; ∆AICc, difference in AICc of the best model. Models are ranked according to their Akaike weight (weight). Sampling year was included as a random effect in models of the *dsx* mimetic allele frequencies. The sampling period (n = 16) was included as a random effect in models of beak mark rate and the number of spermatophores.
